# Transcriptome sequencing revealed that lymph node metastasis of papillary thyroid microcarcinoma is associated with high THBS4 expression and PDGFRA+ cancer-associated fibroblasts

**DOI:** 10.3389/fonc.2025.1536063

**Published:** 2025-04-15

**Authors:** LeYin Hu, Yi Lin, JingYu Zheng, Li Wan, Rui Zhao, Yi Ma, JianMin Li

**Affiliations:** ^1^ Department of Pathology, Wenzhou Medical University First Affiliated Hospital, Wenzhou, Zhejiang, China; ^2^ Department of Pathology, Sanmen People’s Hospital, Taizhou, Zhejiang, China; ^3^ Department of Gastroenterology, Wenzhou Medical University First Affiliated Hospital, Wenzhou, Zhejiang, China

**Keywords:** papillary thyroid microcarcinoma, tumor immune microenvironment, thrombospondin-4, lymph node metastasis, cancer-associated fibroblasts subsets

## Abstract

**Background:**

Cervical lymph node metastasis is a major factor influencing recurrence after surgery for papillary thyroid cancer. Molecular markers that can predict the presence of lymph node metastasis and assess the aggressiveness of papillary thyroid microcarcinoma (PTMC) remain poorly understood. The research question addressed whether specific genes, such as thrombospondin-4 (THBS4), could serve as predictive biomarkers for guiding surgical strategies, particularly in cases where current imaging modalities fail to detect LNM in the central region, and the decision for prophylactic central neck dissection remains controversial.

**Methods:**

Transcriptome sequencing was employed to screen for differentially expressed genes and perform enrichment analysis. The study defined two groups of PTMC patients: LNM(n=50) and NLNM(n=50). 10 samples from each group were used for transcriptome sequencing. The expression of THBS4 was evaluated in both groups. Additionally, the correlation between THBS4 expression and cancer-associated fibroblasts (CAFs), specifically the PDGFRA+ inflammatory CAFs, was investigated to understand the stromal regulatory protein’s role in PTMC aggressiveness.

**Results:**

The analysis of sequencing data revealed that THBS4 expression was significantly higher in LNM PTMC compared to the NLNM group (Fold Change > 1.6 and P < 0.05). LNM PTMCs were also associated with a higher presence of PDGFRA+ inflammatory CAFs (P < 0.05), while no significant difference in the quantity of SMA+ myofibroblastic CAFs was observed between the two groups(P>0.05). Immunohistochemical analysis demonstrated increased THBS4(P < 0.01) and PDGFRA(P < 0.001) expression in LNM groups, while SMA staining showed no significant intergroup differences(P>0.05).

**Conclusion:**

This study’s findings indicate that THBS4 could be a potential biomarker for predicting the risk of lymph node metastasis in papillary thyroid microcarcinoma, thus potentially guiding more personalized surgical interventions. Further validation in larger patient cohorts and the interactions between THBS4 and CAFs are necessary.

## 1 Introduction

Thyroid cancer (TC) is the most prevalent endocrine malignancy and has seen a striking rise in global incidence rates (1). Within TC, differentiated thyroid cancer (DTC) constitutes the majority of cases, with papillary thyroid cancer (PTC) being the most frequent subtype (2). Papillary thyroid carcinoma is prone to cervical lymph node metastasis, and extensive cervical lymph node metastasis also occur in papillary thyroid microcarcinoma (PTMC, traditionally defined as PTCs ≤ 1.0 cm in size), especially in some high-risk histologic subtypes (3). Rationally standardized cervical lymph node dissection is the primary treatment modality for these patients. However, Current imaging techniques, such as ultrasound, lack the sensitivity to reliably detect LNM, particularly in zone VI or in small lymph nodes (4, 5). This limitation hinders clinicians’ ability to effectively identify metastatic lymph nodes. The need for prophylactic lymph node dissection, especially central neck dissection (CND), in these patients in whom no lymph node metastasis is detected at preoperative examination is still controversial in various guidelines (6–9). The benefits of preventive CND remain unclear. Therefore, effective identification of cervical lymph node metastasis is an urgent issue to be explored. Nonetheless, a subset of PTMCs presents with adverse pathologic features and aggressive clinical behaviors, including lymph node metastasis, distant metastasis, and structural recurrence following surgery (10–13). In severe instances, these tumors can be fatal, with progression to high-grade carcinoma often observed in metastatic lymph nodes (14, 15).

To address these diagnostic challenges, molecular diagnostic techniques are increasingly used to complement radiographic examinations. Although BRAFV600E is the most common mutation in papillary thyroid cancer, its role as a reliable risk factor for lymph node metastasis is yet to be determined (16, 17). Hence, there is a pressing need to identify new molecular markers that can predict the likelihood of lymph node metastasis.

Advances in high-throughput sequencing technology have facilitated the discovery of molecular markers for PTMCs. Despite recent advances (18–21), the genomic differences between PTMC with and without lymph node metastasis remain underexplored. In this study, we employed transcriptome sequencing to investigate the genomic characteristics of PTMC, identifying genomic features that diverge from previous reports. After performing further screening we found that thrombospondin-4 (THBS4) and its corresponding proteins were associated with whether PTMC developed lymph node metastasis or not. AS an extracellular matrix protein, THBS4 is usually considered to play a key role in tissue growth and remodeling under physiological conditions (22–24). In the context of tumor biology, THBS4 may contribute to tumor growth, proliferation, and migration, thereby promoting aggressive tumor behavior (25–27). Notably, THBS4 expression in some tumors is not directly secreted by tumor cells but rather by cancer-associated fibroblasts (CAF) (27–29). For this reason, we further explored the correlation between THBS4 and CAF in PTMC. Our results suggest THBS4 may serve as a molecular marker for predicting lymph node metastasis in PTMC.

## 2 Materials and methods

### 2.1 Biospecimen collection, pathological assessment, and public data processing

This retrospective study was approved by local ethical committees (The First Hospital of Wenzhou Medical University), and written informed consents were obtained from all patients. FFPE tissue samples (stored within six months) and corresponding haematoxylin-eosin stained slides from 100 PTMC patients were obtained from the Department of Pathology, First Affiliated Hospital, Wenzhou Medical University, and 20 of these were used for RNA sequencing. We performed simple random sampling using R (v4.0.3) with the command set.seed(); selected_LNM<-sample(1:50, size=10, replace=FALSE), selected_NLNM<- sample(51:100, size=10, replace=FALSE).Two pathologists independently performed histopathological review of the tumor sections. TNM stage of the disease was defined by pathologists according to the 8th AJCC/UICC staging system. All enrolled patients with thyroid carcinoma met the following inclusion criteria (1): Primary tumor;(2) Maximum tumor diameter ≤1 cm;(3) Histologically confirmed papillary thyroid carcinoma;(4) At least central neck dissection performed during surgery;(5) Surgical excision of suspicious regional lymph nodes identified during preoperative evaluation;(6) Absence of distant metastasis (M0). RNA-sequencing counts, and clinical data of 48 PTC and normal thyroid tissue were acquired from The Cancer Genome Atlas Program (https://portal.gdc.cancer.gov/projects/TCGA-THCA). Use ComBat and ComBat_seq from the SVA package to correct for batch effects.

### 2.2 Nucleic acid extraction and library construction

Total RNA from FFPE samples was extracted using miRNeasy FFPE kit (QIAGEN). Ribosomal RNA was depleted using KAPA Stranded RNA-seq Kit with RiboErase (HMR) (KAPA Biosystems). Library preparations were performed with KAPA Stranded RNA-seq Library Preparation Kit (Roche). Library concentration was determined by KAPA Library Quantification Kit (KAPA Biosystems), and library quality was accessed by Agilent High Sensitivity DNA kit on Bioanalyzer 2100 (Agilent Technologies), which was then sequenced on Illumina Novaseq6000 NGS platforms (Illumina).

### 2.3 Gene expression analysis and sequent analysis

Base calling was performed on bcl2fastq v2.16.0.10 (Illumina, Inc.) to generate sequence reads in FASTQ format (Illumina 1.8+ encoding). Quality control (QC) was performed with Trimmomatic (version 0.33) (30). STAR (version 2.5.3a) (31) is used for transcriptome mapping followed by isoform and gene level quantification performed by RSEM (version 1.3.0) (32). Differential expression analysis was conducted by R packages DESeq2 (version 1.16.1) (33) and edgeR (version 3.18.1) (34). Differentially expressed genes of cohort were selected by Fold Change > 1.6 and P value < 0.05. Data from one of the samples was removed as an outlier. Differentially expressed genes of TCGA dataset were selected by Fold Change > 2 and P value < 0.05. Corresponding volcano plots and heatmaps were generated by in-house R scripts. GO and KEGG enrichment analysis were performed by ClusterProfiler (version 3.6.4) (35). Gene set enrichment analyses (GSEA) were performed using the GSEA software. The NMF package was used to perform an NMF clustering. K-M survival curves coupled with Logrank test were performed using the R packages “survival” (v.3.4–0) and “survminer” (v.0.4.9). The relative abundance of immune cell populations was then calculated using the R package “immunedeconv” (v.2.0.4) (36), which allows the community to perform integrated deconvolution using seven approaches including xCell (37) (Detailed data are provided in the **Supplementary Material**). Receiver operating characteristics (ROC) analysis was performed using the R package pROC (version 1.17.0.1) to obtain AUC.

### 2.4 Survival analysis

We downloaded the harmonized and standardized pan-cancer dataset from the UCSC (https://xenabrowser.net/) database and the previously published TCGA prognostic study in Cell (38). Prognostic dataset from UCSC (https://xenabrowser.net/datapages/), and TARGET follow-up data from UCSC (https://xenabrowser.net/datapages/) As a supplement, samples with expression level of 0 were filtered and samples with follow-up shorter than 30 days were excluded, and a log2(x+0.001) transformation was performed for each expression value to exclude cancers with less than 10 samples in a single cancer type. We calculated the optimal cut-off value of ENSG00000113296(THBS4) using the R package maxstat, and set the minimum number of group samples to be greater than 25% and the maximum number of group samples to be less than 75% to finally obtain the optimal cut-off value, based on which the patients were divided into high and low groups, and further analyzed the prognostic difference between the two groups using the survfit function of the R package survival, and the significance of the prognostic difference between the samples of different groups was assessed using the logrank test method. The prognostic differences between the two groups were further analyzed using the survfit function of the R package survival, and the significance of the prognostic differences between the samples of different groups was assessed using the logrank test method.

### 2.5 Immunohistochemistry staining and quantification

Immunohistochemistry was performed. Briefly, sections were dewaxed and rehydrated. Antigen retrieval was performed by pretreating the slides in citrate buffer (pH 6.0; Thrombospondin 4) in a pressure cooker for 1 minute or EDTA (pH 8.0; PDGFRA and SMA) boiling for 20 minutes at 95°C. The slides were incubated with PBS containing 3% hydrogen peroxide for 10 min and subsequently incubate in the primary antibody (dilution ratios 1:150, Thrombospondin 4 antibody, catalog number orb1289935, biorbyt; dilution ratios 1:50, PDGFRA antibody, catalog number ZA-0377, ZSGB-BIO; dilution ratios 1:200, SMA antibody, catalog number ZM-0003, ZSGB-BIO) at 40°C for 1 hour. The slides were then probed with horseradish peroxidase conjugated secondary antibody for 20 mins at 40°C, followed by reaction with diaminobenzidine and counterstaining with Mayer’s hematoxylin. Immunohistochemistry sections were digitally scanned using a whole slide image scanner. FIJI software was utilized for the quantitative assessment of average density within the region of interest.

### 2.6 Statistical analysis

All analyses were performed using R software v4.0.3 (https://cran.r-project.org/). T test was used to compare the distributions of continuous variable. Then chi-square test was performed to compare the composition differences of categorical variable. Correlation was obtained with Spearman correlation test. Unless otherwise noted, a p-value < 0.05 was considered statistically significant.

## 3 Result

### 3.1 Research process and clinicopathologic features

**Figure 1:**
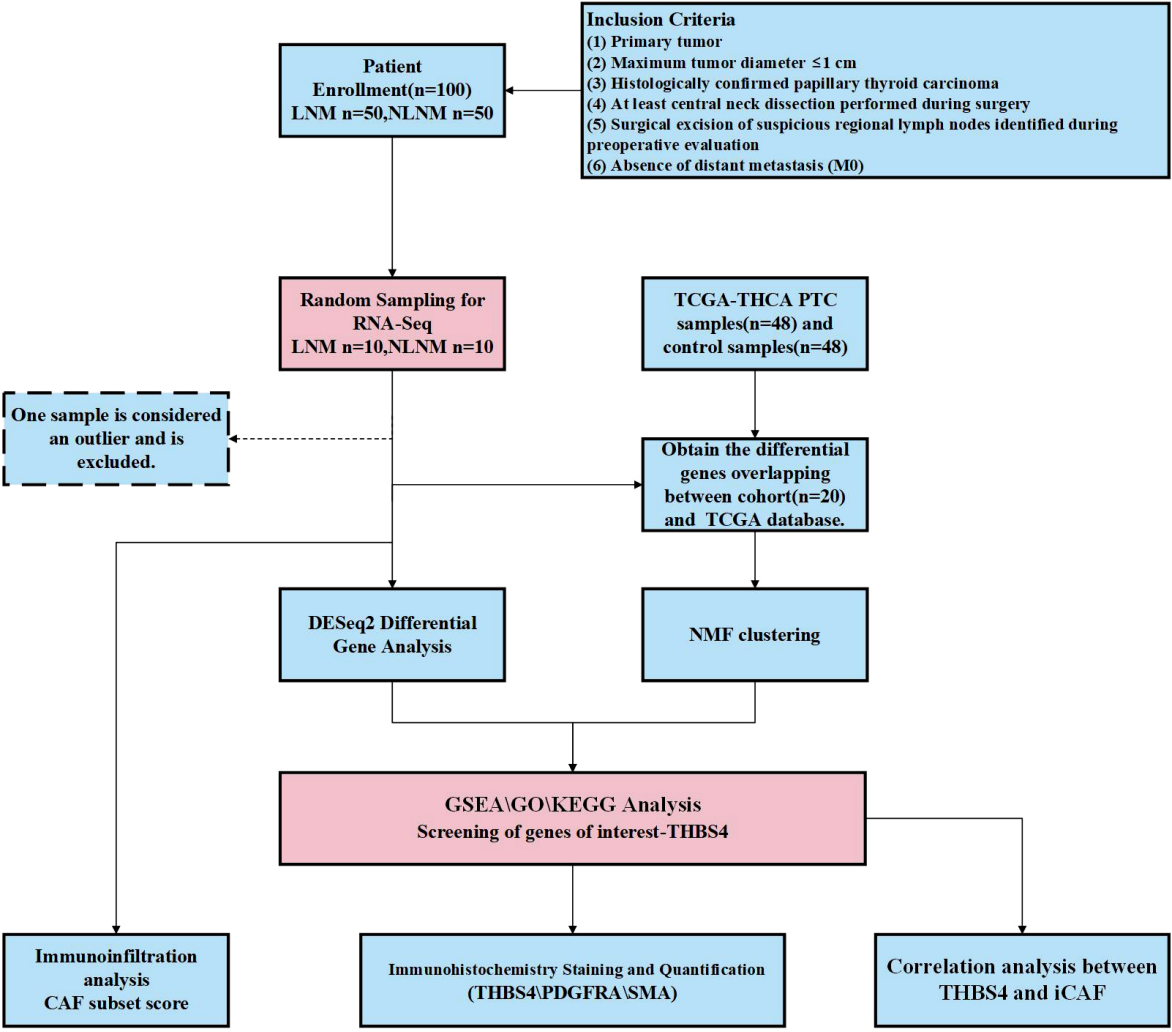
The flowchart of this study. PTC, papillary thyroid carcinoma; LNM. lymph node metastasis; NLNM, non-lymph node metastasis; THBS4, thrombospondin-4; CAF, cancer-associated fibroblasts; iCAF inflammatory CAF.

The process of this study is shown in **Figure 1**. We defined PTMC as two groups; those with pathologically confirmed at least one regional lymph node metastasis, which we termed the LNM group, and those without, termed the NLNM group. There were no significant differences in baseline characteristics between the two groups (**Table 1**).

**Table 1:** Patients’ clinicopathologic features.

Variable	NLNM(n=50)	LNM(n=50)	*P*-value
Age(n)			0.288
≥55 years	19	14	
<55 years	31	36	
Gender(n)			0.317
Male	27	22	
Female	23	28	
Maximum diameter of nodule (mm)	0.640 ± 0.176	0.680 ± 0.173	0.256
T stage(n)			1.000
T1a	50	50	
Minimal Extrathyroid Extension			0.110
No	47	42	
Yes	3	8	
BRAF mutation(n)			0.487*
Yes	44	47	
No	6	3	
Subtype(n)			0.827
Classic	27	26	
Infiltrative follicular	18	17	
Tall cell	5	7	

Statistical analysis was performed using chi-square test or Fisher’s exact test* for categorical variable and t test for continuous variable

### 3.2 Differential gene expression analysis between LNM and NLNM PTMC

**Figure 2:**
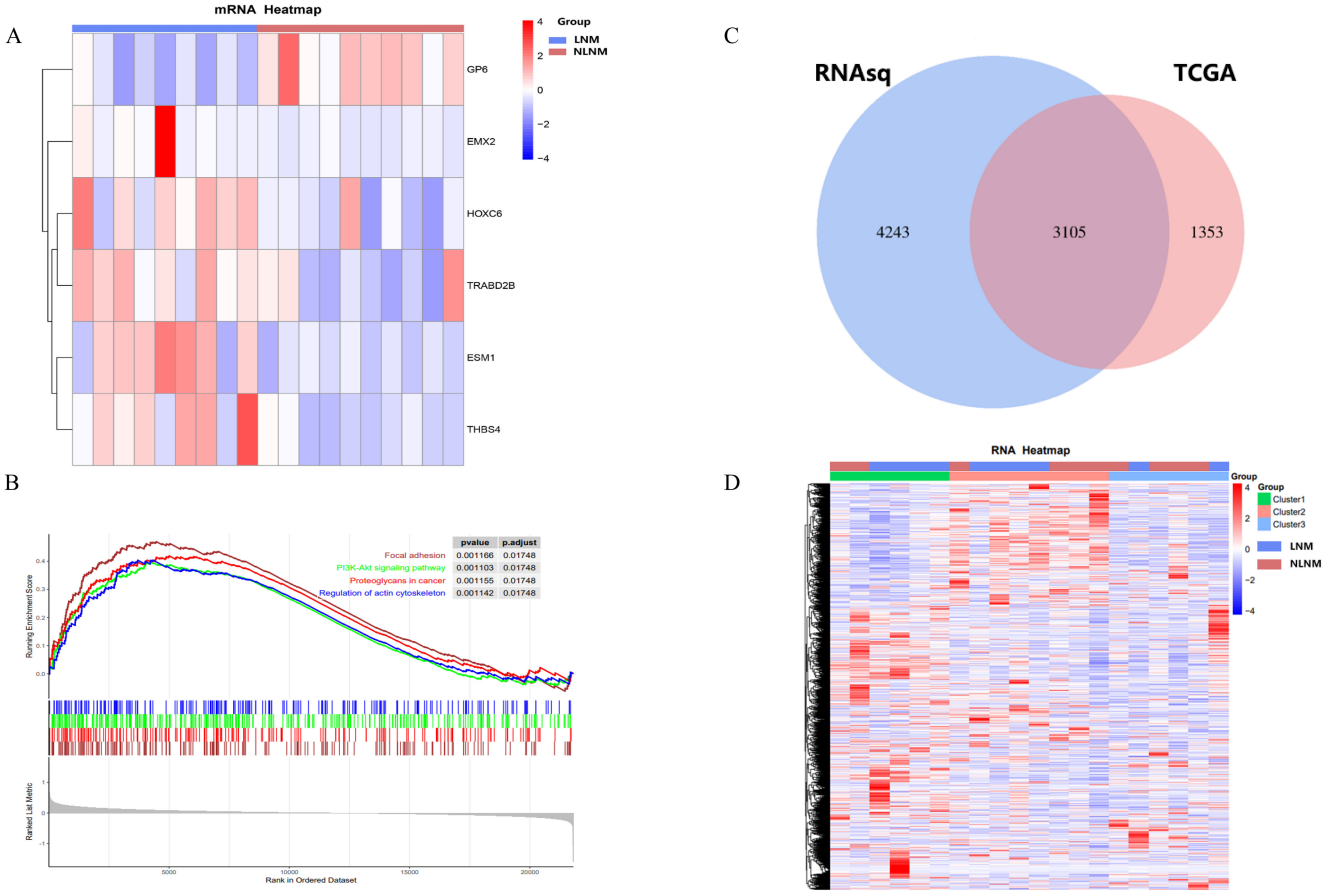
**(A)** Heatmap of differentially expressed genes encoding messenger RNAs. **(B)** The Gene Set Enrichment Analysis (GSEA) of all DEGs. **(C)** DEGs in the two cohorts and **(D)** NMF clustering of differentially expressed genes.

**Figure 3:**
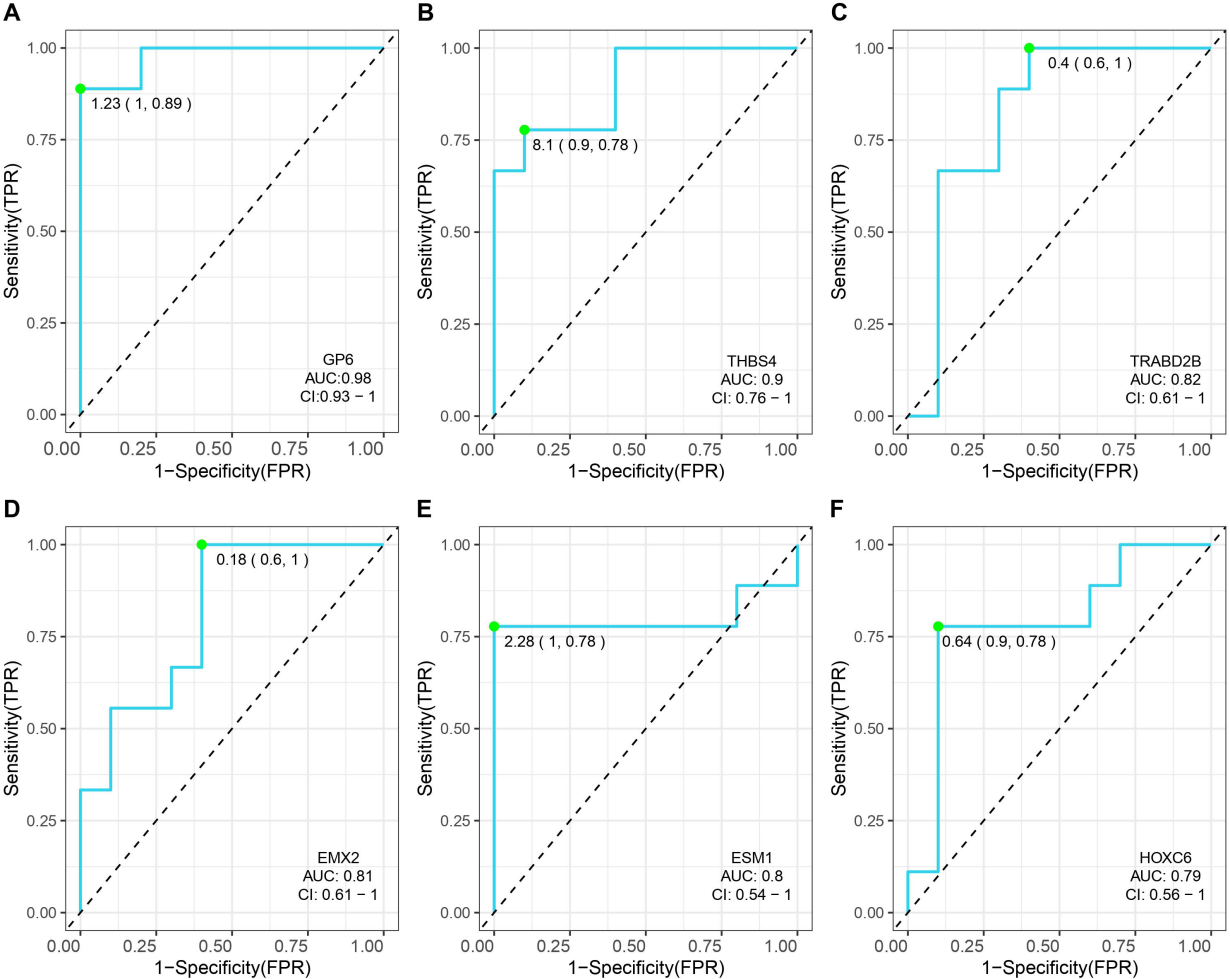
Receiver operating characteristic (ROC) curves and the associated areas under curves (AUCs) of six gene for the PTMC cohort. **(A)** GP6,glycoprotein VI platelet; **(B)** THBS4,thrombospondin 4; **(C)** TRABD2B,TraB domain containing 2B; **(D)** EMX2,empty spiracles homeobox 2 **(E)** ESM1,endothelial cell specific molecule 1; **(F)** HOXC6,homeobox C6;

**Figure 4:**
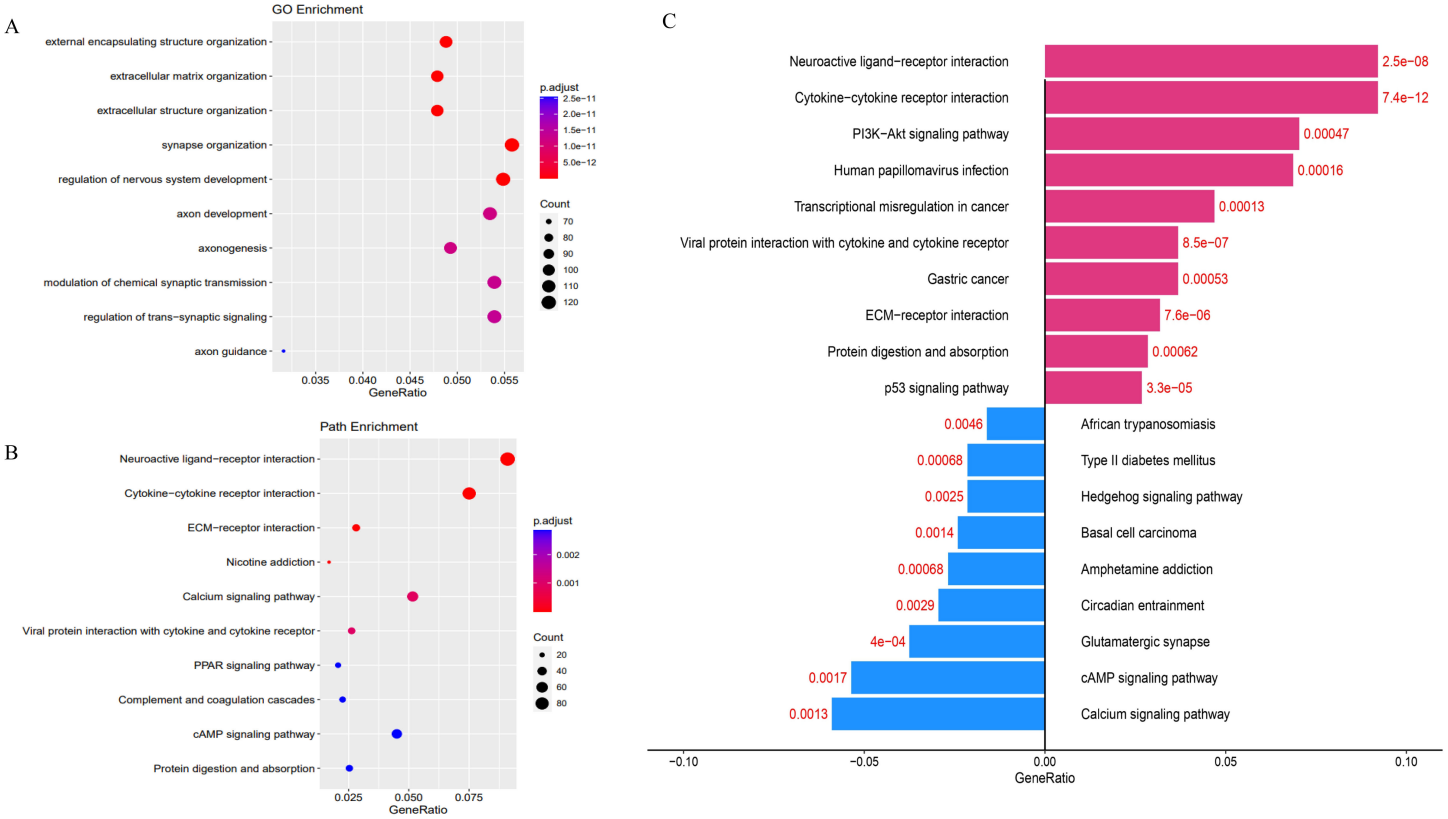
**(A)** Gene Ontology (GO) and **(B)** KEGG analysis of DEGs in two cohorts. **(C)** Gene Ontology analysis of DEGs in cluster 1.

**Figure 5:**
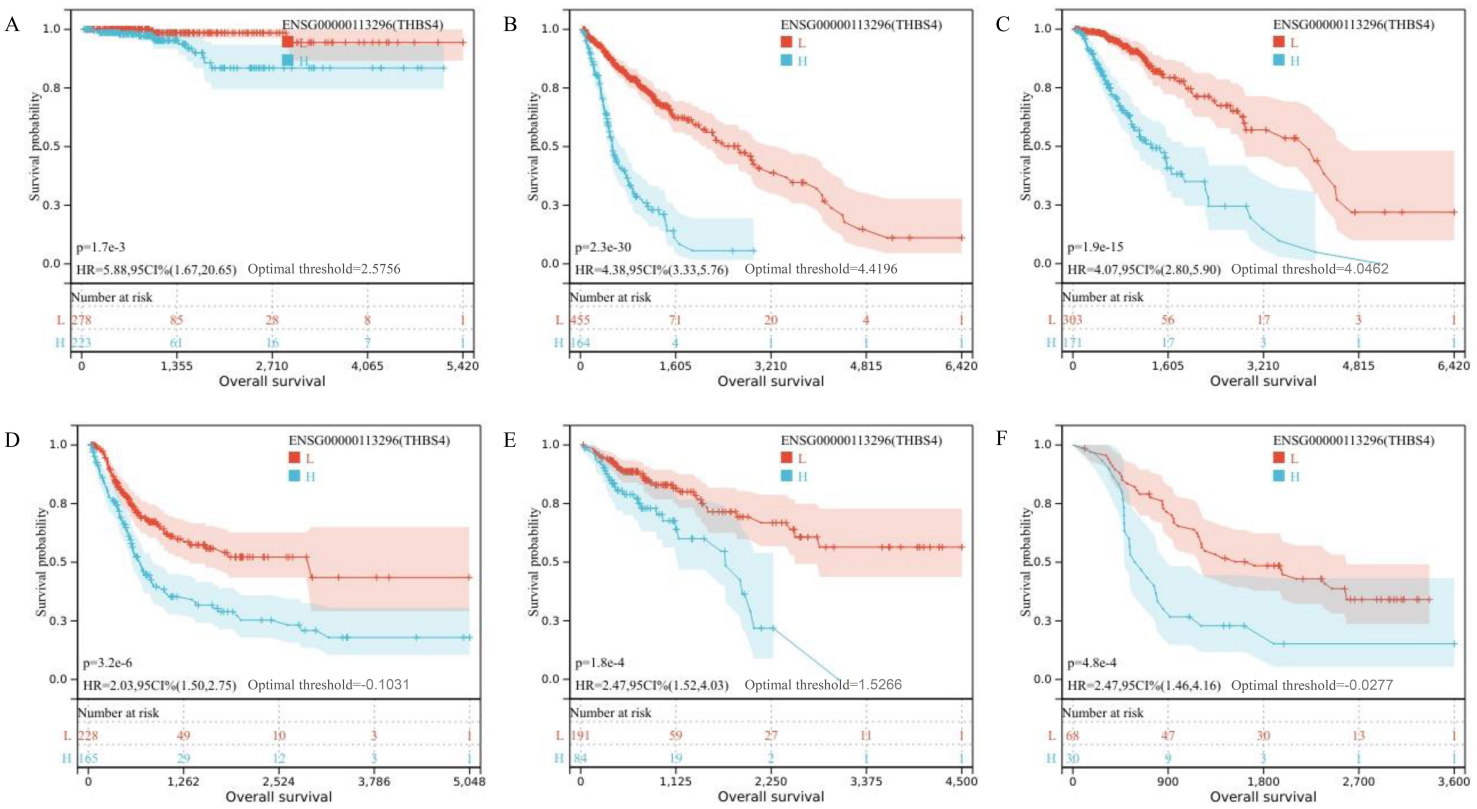
**(A–F)** Kaplan-Meier survival analysis showing overall survival for TCGA-THCA, TCGA-GBMLGG, TCGA-LGG, TCGA-BLCA, TCGA-COAD and TCGA-ALL-R.

**Figure 6:**
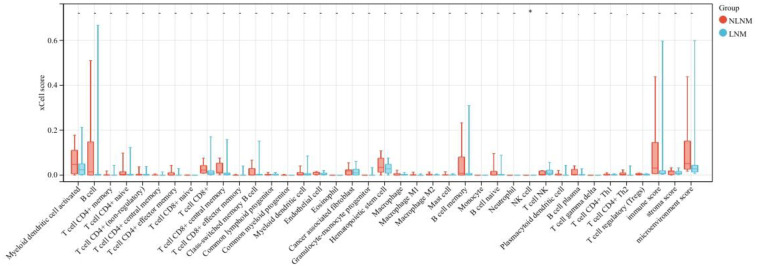
Differences in infiltrating immune cells between LNM and NLNM groups.

Transcriptome sequencing revealed 61 differentially expressed genes (DEGs) between the two groups. 55 of these were long non-coding RNAs and 6 were messenger RNAs (**Figure 2A**, **Supplementary Table S2**). Subsequently, Gene Set Enrichment Analysis (GSEA) was conducted on all expressed genes, revealing significant enrichment in 13 pathways. Notably, these pathways encompass focal adhesion, the PI3K-Akt signaling pathway, proteoglycans in cancer, and regulation of the actin cytoskeleton (**Figure 2B**). ROC analysis showed good differentiation of the two tumor groups by expression level of GP6 and THBS4 (**Figure 3**). To further screen for genes of interest, we obtained RNA sequencing data and clinical information from the TCGA-THCA dataset for PTC, selecting 48 cases each of tumor samples and normal thyroid samples. Using the normal thyroid tissue transcriptome from TCGA as a control, we identified DEGs between tumor and normal tissues separately. Our cohort data revealed 7348 DEGs and TCGA 4458 DEGs. Among the 3105 overlapping genes, 1746 were simultaneously upregulated and 1078 were simultaneously downregulated in both cohorts (**Figures 2C, D**). These DEGs were then subjected to Gene Ontology (GO) and Kyoto Encyclopedia of Genes and Genomes (KEGG) analysis (**Figures 4A, B**), which highlighted significant effects on extracellular matrix organization and ligand-receptor interactions, including extracellular matrix (ECM)-receptor interaction. Non-negative Matrix Factorization (NMF) clustering grouped the 20 samples into three clusters, with clusters 1 exhibiting a higher proportion of LNM tumors (**Figure 2D**). Next, we performed an enrichment analysis of cluster 1 and showed that pathways such as PI3K-Akt signaling pathway and ECM-receptor interaction were up-regulated (**Figure 4C**). We analyzed the impact of THBS4 on the prognosis of patients with THCA and several other tumors using the TCGA database. Kaplan-Meier survival analyses showed that the higher THBS4 expression group typically had worse overall survival (**Figure 5**). These results suggest that tumor impact on the mesenchyme may significantly influence the probability of biological behavior of tumors including lymph node metastasis. We therefore selected THBS4, a gene involved in PI3K-Akt signaling pathway and associated with mesenchymal components, for further exploration. Additionally, we compared the abundance of immune cells in the microenvironment of both groups using seven common algorithms. In our cohort, the two groups did not differ significantly in the composition of the immune microenvironment, with only differences observed in the abundance of NK cells (**Figure 6**).

### 3.3 THBS4 is highly expressed in LNM PTMC

**Figure 7:**
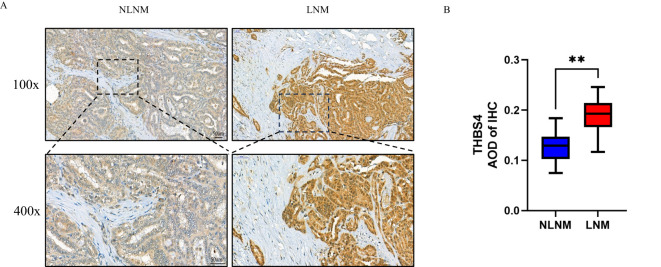
**(A)** Representative image of the immunohistochemistry of THBS4. **(B)** Average optical density (AOD) of immunohistochemical staining image. **P < 0.01 by Wilcoxon rank-sum test.

THBS4, one of the genes we identified, is involved in the focal adhesion and PI3K-Akt signaling pathways and is highly expressed in the LNM group. According to recent studies, the origin of THBS4 varies in different tumors, either from tumor cells or mesenchymal stromal cells, suggesting heterogeneity of THBS4 expression among tumors (29, 39). Immunohistochemical analysis of THBS4 expression in LNM and NLNM PTMC tissues revealed more positive staining in the tumor cytoplasm of the former, while fibroblasts in the tumor mesenchyme were barely stained (**Figures 7A, B**).

### 3.4 Increased abundance of PDGFRA+CAFs in LNM PTMC

In some studies, THBS4 regulates tumor biological behavior by affecting CAF (27), whereas a similar process has not been intensively investigated in papillary thyroid carcinoma. CAF is usually the most abundant mesenchymal cell component and may play a number of important biological functions in papillary thyroid carcinoma. Through single-cell sequencing, several studies have identified more functional subsets of CAF in various tumors (40–42). As with papillary thyroid carcinoma, CAFs have been broadly categorized into myofibroblastic CAFs (myoCAFs) and inflammatory subset (iCAFs) (43, 44). So, we next assessed the abundance of both CAFs in our cohort.

**Figure 8:**
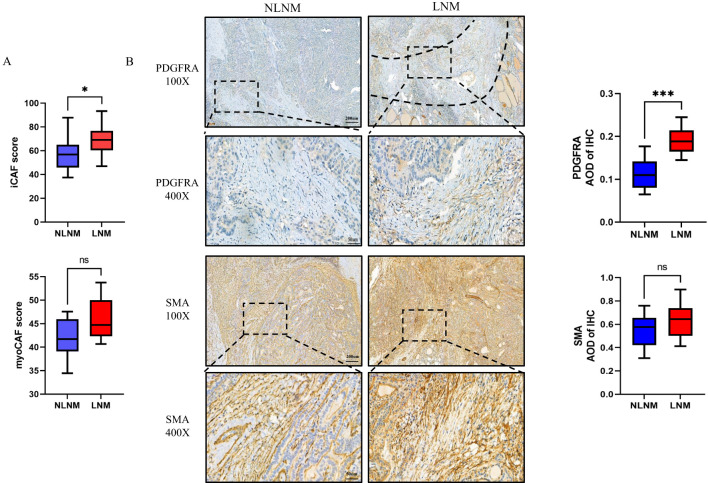
**(A)** Marker gene scores for CAF subsets in the LNM and NLNM groups. p=0.0433 or p=0.0753 by Wilcoxon rank-sum test. **(B)** Representative image of immunohistochemistry of PDGFRA and SMA, and corresponding average optical density (AOD). The dotted line shows the fibrous band between the tumor and normal tissue *P < 0.05 or ***P < 0.001 by Wilcoxon rank-sum test. ns, no significance.

We assessed the abundance of both CAF subsets in our cohort, using marker gene expression averages derived from single-cell sequencing, as in previous studies (45) (**Supplementary Table S1**). We found that myoCAF scores did not differ between the two groups, whereas iCAF scores were higher in the LNM tumors, suggesting an increased presence of iCAFs in these cases (**Figure 8A**). Considering that there is no exclusive marker for either subset, after referring to previous studies (44), we used SMA and PDGFRA to label myoCAF and iCAF, respectively, and immunohistochemical staining showed an abundant presence of myoCAFs in both groups, while iCAFs were relatively scarce and primarily located at the infiltrating leading edge of the tumor in LNM PTMC (**Figure 8B**). Also, A significant positive correlation was observed between THBS4 expression and iCAF scores (**Supplementary Figure S1**. R=0.738, p<0.001, Spearman rank correlation).

## 4 Discussion

In our study, we investigated the genomic disparities between PTMC with and without LNM, yielding new insights through transcriptome sequencing. Though the six mRNAs (TRABD2B, GP6, THBS4, ESM1, HOXC6, EMX2) lack direct experimental evidence of interaction in our study, emerging literature suggests their potential convergence in metastatic progression. THBS4 and ESM1 may cooperatively modulate TGF-β bioavailability—a known EMT inducer—and endothelial activation (23, 46). EMX2 has been implicated in epithelial plasticity regulation via epigenetically silenced (47, 48). As a platelet-specific collagen receptor, GP6 is known to mediate platelet adhesion and activation, which may indirectly influence tumor progression through platelet-tumor microenvironment crosstalk (49). Intriguingly, HOXC6 might participate in stromal reprogramming (50) and stem cell identity maintenance (51). While this hypothetical framework requires experimental validation, the genes’ collective enrichment in “extracellular matrix organization” and “ECM-receptor interaction” implies a plausible biological synergy warranting further investigation.

From the DEGs identified in our discovery cohort, THBS4 emerged as the prime candidate for three synergistic reasons (1): Its expression exhibited superior diagnostic accuracy in distinguishing LNM from NLNM tumors (AUC=0.9), outperforming other candidates; (2) Functional triangulation through GSEA, TCGA cohort validation, and NMF clustering convergently implicated PI3K-Akt signaling and ECM remodeling – pathways directly modulated by THBS4; (3) Pan-cancer survival analysis revealed that THBS4 overexpression universally predicted poor prognosis, suggesting its conserved role in metastatic progression. We therefore propose THBS4 as a promising marker gene for predicting lymph node metastasis and for guiding surgical strategies, yet the precise mechanisms by which it influences PTMC behavior remain unclear. We hypothesized that THBS4 may alter the composition of CAF subsets in the PTMC mesenchyme, and our findings support the notion of increased iCAF presence in LNM group. These discoveries enhance our comprehension of PTMC.

Previous studies have provided some characterization of PTMC transcriptome features. Fan Yang et al. identified nine core genes that may be used to predict the development of PTMC (18). A group of genes, including collagen type I alpha 1 (COL1A1), fibronectin 1 (FN1), laminin subunit gamma 2 (LAMC2), periostin (POSTN), transforming growth factor beta induced (TGFBI), are involved in extracellular mesenchymal organization and affect tumor behavior, akin to our findings. However, the impact of these genes on lymph node metastasis was not investigated. Other studies compared the transcriptome characteristics of patients with or without lateral lymph node metastasis. Consistent with our study, the differences in genetic characteristics between the two groups were relatively modest (19). Dilmi Perera et al. identified several DEGs, but they do not share a common molecular pathway or a single gene expression profile, making it difficult to explain the underlying physiological processes that the occurrence of LNM (20). The role of epithelial-mesenchymal transition and cancer stem cell-like properties in extensive lymph node spread of PTMC has been noted, as have the associations of lncRNA and circRNA with LNM (52–54). Overall, these studies have primarily focused on tumor parenchymal cells in search of markers predicting adverse behavior. A recent large sample study categorized PTMC into PTMC-proliferation (PTMC-Pro) and PTMC-inflammatory (PTMC-Inf) types based on their respective marker genes (55). PTMC- Inf was related with activated immune cell signaling and interferon-γ response and had a lower 5-year PFS rate than PTMC-pro. In contrast to our study, there were more differences in immune cell abundance between PTMC-Pro and PTMC-Inf. This suggests that in addition to clinical features, PTMC are heterogeneous in immune microenvironment, potentially leading to early-stage extensive lymph node metastasis. Our study, therefore, centered on tumor-microenvironment interactions, particularly the roles of THBS4 and CAFs.

Thrombospondin-4 (THBS-4) is a member of the thrombospondin protein family, which consists of five highly homologous members. In human tissues, THBS4 is abundantly expressed in cardiovascular, skeletal muscle, tendon and nerve tissues, with roles in cardiovascular and skeletal muscle being the most extensively studied. In the healthy heart, THBS4 prevents interstitial ECM deposition and cardiac hypertrophy (56, 57). And in skeletal muscle, THBS4 is important for proper motor unit assembly and function, and both muscle and tendon require THBS4 for attachment (58). *In vitro*, experiments have confirmed that THBS4 inhibits collagen synthesis in human fibroblasts and endothelial cells. All of these functions may be related to the regulation of ECM by THBS4, and THBS4 deficiency results in cardiac interstitial ECM deposition and skeletal muscle ECM deficiency.

Recently, high expression of THBS4 was found in several tumors. Interestingly, in most tumors, THBS4 promoted tumor progression. Similar to our findings in PTMC, in hepatocellular carcinoma, bladder cancer and prostate cancer, high expression of THBS4 in tumor cells promoted tumor growth, proliferation and invasion (25, 26, 59). In contrast, in colon cancer, the THBS4 gene is methylated and silenced, while increased expression of THBS4 in colon cancer colonies significantly inhibits tumor growth (60). The role of THBS4 on tumors is not only complex, but also varies in its origin in tumors. A typical example is gastric adenocarcinoma where, in contrast to PTMC, THBS4 is derived from tumor-associated fibroblasts in diffuse gastric adenocarcinoma, whereas THBS4 expression is not detectable in tumor cells (28, 29, 61). Another study in gallbladder cancer found that THBS4 is secreted by a variety of cells, but its main source is also CAF (27); in addition to CAF, the abundance of tumor-associated macrophages can also be regulated by THBS4 and play a role in tumor invasiveness (62). The specific regulatory mechanism of THBS4 in both normal and tumor tissues is not well understood, among which transforming growth factor β (TGF-β) signaling has been shown to be closely related, and it has been reported that SMAD3 is involved in the regulation of THBS4 by TGF-β (23).

The tumor microenvironment (TME) is a concept that has emerged in recent years and is defined as the surrounding microenvironment in which tumor cells exist, including peripheral blood vessels, immune cells, fibroblasts, myeloid-derived inflammatory cells, a variety of signaling molecules, and extracellular matrix (ECM). In current studies, CAF is typically one of the most abundant components of the TME (63). Some studies have shown that CAF is associated with LNM, upregulation of immune checkpoints, enrichment of immune cells, and tumor-associated macrophage polarization in PTCs (64). However, the heterogeneity of CAF in TME was not well recognized in PTMC until the widespread use of single-cell sequencing technology. Previous studies of PTC have used only SMA-labeled CAF, leaving the impact of CAF subsets on tumors poorly understood (65). A recent multi-sample single-cell sequencing study revealed distinct subsets of CAF in PTC (44). One subset is associated with cell motility, contraction, and extracellular matrix, and the other subset is associated with an abundance of immunomodulatory molecules and chemokines. iCAF is often hypothesized to carry out intercellular communication to influence the biological behavior of tumors, which may consequently undergo adverse pathologic features (66–68). Another single-cell sequencing of a single sample yielded similar results (43). In other tumors, CAF isoforms exert immunomodulatory functions through the expression of various cytokines, a typical molecule being interleukin-6 (69, 70). However, the situation in PTC is somewhat different. Weilin Pu et al. hypothesized through bioinformatics analysis that myoCAF tends to exert mechanical and chemical influences on tumor progression, whereas iCAF exerts immunomodulatory functions by recruiting and crosstalking various immune cells through chemokines such as CCL5, CCL3L3 and other chemotactic factors in the TME (44).

To our knowledge, no biological validation of the CAF subset in PTMC has been performed. Here, we compared data from samples from our center with previous studies, similar to iCAF with less cellular abundance than myoCAF, only iCAF differed between LNM and NLNM PTMC, and the expression level of THBS4 was positively correlated with the iCAF score. Based on previous studies and our results, we speculate that tumor cells and CAF in PTMC may be involved in bidirectional regulation through THBS4 and TGF-β signaling pathways, which needs to be further confirmed in *in vitro* and *in vivo* experiments. In addition, in the available bulk RNA sequencing study, CAF did not show an effect on the biological behavior of PTC, probably because iCAF accounted for a small proportion of the total CAF (55).

Our study demonstrates the potential clinical utility of using THBS4 and PDGFRA as biomarkers to predict PTMC lymph node metastasis. Regrettably, there are several limitations to this study. First, this is a single-center study, and the generalizability of its results needs to be validated by a larger cohort. Second, we did not analyze the prognosis of patients in this cohort because of the short follow-up time. Thirdly, although no significant differences were observed in baseline patient characteristics, certain selection biases existed in the histological subtype selection of our cohort. This limitation was primarily attributable to the exclusion of rare histological subtypes from our study population, and secondly, to the inherent propensity of certain subtypes to exhibit higher rates of lymph node metastasis (3). Furthermore, in the AJCC 8^th^ edition, extrathyroid extension that is only visible microscopically does not increase the T stage because it does not affect the patient’s prognosis (71). However, tumor invasion of the vasculature or nerves in the soft tissue can sometimes be seen. Although it is unclear whether this leads to enhanced metastatic potential, this pathological categorization may nevertheless introduce potential confounding factors. In addition, we did not distinguish the pattern of lymph node metastases in detail, which may have introduced confounding bias. Whether different metastatic patterns represent different underlying mechanisms remains to be investigated in larger samples and cohorts. Finally, we did not investigate the causal relationship between THBS4 expression and iCAF abundance in tumors. We were unable to provide evidence of a direct association between THBS4 and iCAF. Further mechanistic studies are essential to elucidate the exact interaction between THBS4 and iCAF and to identify potential therapeutic targets.

In conclusion, our study integrated transcriptome sequencing and TCGA RNA sequencing data and identified THBS4 as a potential biomarker for predicting lymph node metastasis in papillary thyroid microcarcinoma (PTMC). Our findings suggest that THBS4 expression levels may influence the composition of CAF subsets in the tumor microenvironment. Despite these promising results, our study has limitations, including its single-center design and the need for further studies on the interaction between THBS4 and iCAFs. Future studies should validate these findings in larger cohorts and explore the predictive value of THBS4 and PDGFRA as biomarkers of PTMC lymph node metastasis.

## Data Availability

The datasets generated in this study are available through the GSA for Human in the Genome Sequence Archive (GSA), BioProject ID: PRJCA038155, accession ID: HRA011009. The data of the public database can be accessed through the above-mentioned URLs.

## Supplementary material

Supplementary Figure 1 Correlation analysis of expression of THBS4 and iCAF scores, R=0.738, p<0.001, Spearman rank correlation

## References

[1] Sung H Ferlay J Siegel RL Laversanne M Soerjomataram I Jemal A. Global cancer statistics 2020: GLOBOCAN estimates of incidence and mortality worldwide for 36 cancers in 185 countries. CA A Cancer J Clin. (2021) 71:209–49. doi: 10.3322/caac.21660 33538338

[2] Schmidbauer B Menhart K Hellwig D Grosse J. Differentiated thyroid cancer—Treatment: state of the art. IJMS. (2017) 18:1292. doi: 10.3390/ijms18061292 28629126 PMC5486113

[3] Baloch ZW Asa SL Barletta JA Ghossein RA Juhlin CC Jung CK. Overview of the 2022 WHO classification of thyroid neoplasms. Endocr Pathol. (2022) 33:27–63. doi: 10.1007/s12022-022-09707-3 35288841

[4] Zhang Y Lu Yy Li W Zhao Jh Zhang Y He Hy. Lymphatic contrast-enhanced US to improve the diagnosis of cervical lymph node metastasis from thyroid cancer. Radiology. (2023) 307:e221265. doi: 10.1148/radiol.221265 37014243

[5] Kim SK Woo JW Park I Lee JH Choe JH Kim JH. Computed tomography-detected central lymph node metastasis in ultrasonography node-negative papillary thyroid carcinoma: is it really significant? Ann Surg Oncol. (2017) 24:442–9. doi: 10.1245/s10434-016-5552-1 27624581

[6] Ito Y Masuoka H Fukushima M Inoue H Kihara M Tomoda C. Excellent prognosis of patients with solitary T1N0M0 papillary thyroid carcinoma who underwent thyroidectomy and elective lymph node dissection without radioiodine therapy. World J Surg. (2010) 34:1285–90. doi: 10.1007/s00268-009-0356-0 20041244

[7] Viola D Materazzi G Valerio L Molinaro E Agate L Faviana P. Prophylactic central compartment lymph node dissection in papillary thyroid carcinoma: clinical implications derived from the first prospective randomized controlled single institution study. J Clin Endocrinol Metab. (2015) 100:1316–24. doi: 10.1210/jc.2014-3825 25590215

[8] Ito Y Higashiyama T Takamura Y Miya A Kobayashi K Matsuzuka F. Risk factors for recurrence to the lymph node in papillary thyroid carcinoma patients without preoperatively detectable lateral node metastasis: validity of prophylactic modified radical neck dissection. World J Surg. (2007) 31:2085–91. doi: 10.1007/s00268-007-9224-y 17885787

[9] jun ZW Luo H mei ZY yu DW qiang ZJ. Evaluating the effectiveness of prophylactic central neck dissection with total thyroidectomy for cN0 papillary thyroid carcinoma: An updated meta-analysis. Eur J Surg Oncol. (2017) 43:1989–2000. doi: 10.1016/j.ejso.2017.07.008 28807633

[10] Yang F Zhong Q Huang Z Lian M Fang J. Survival in papillary thyroid microcarcinoma: A comparative analysis between the 7th and 8th versions of the AJCC/UICC staging system based on the SEER database. Front Endocrinol. (2019) 10:10. doi: 10.3389/fendo.2019.00010 PMC6354565 30733707

[11] Jin WX Ye DR Sun YH Zhou XF Wang OC Zhang XH. Prediction of central lymph node metastasis in papillary thyroid microcarcinoma according to clinicopathologic factors and thyroid nodule sonographic features: a case-control study. CMAR. (2018) 10:3237–43. doi: 10.2147/CMAR.S169741 PMC6130265 30233240

[12] Arora N Turbendian HK Kato MA Moo TA Zarnegar R Fahey TJ. Papillary thyroid carcinoma and microcarcinoma: is there a need to distinguish the two? Thyroid. (2009) 19:473–7. doi: 10.1089/thy.2008.0185 19348582

[13] Park YJ Kim YA Lee YJ Kim SH Park SY Kim KW. Papillary microcarcinoma in comparison with larger papillary thyroid carcinoma in BRAF ^V600E^ mutation, clinicopathological features, and immunohistochemical findings. Head Neck. (2010) 32:38–45. doi: 10.1002/hed.21142 19475551

[14] Ghossein R Ganly I Biagini A Robenshtok E Rivera M Tuttle RM. Prognostic factors in papillary microcarcinoma with emphasis on histologic subtyping: A clinicopathologic study of 148 cases. Thyroid. (2014) 24:245–53. doi: 10.1089/thy.2012.0645 23745671

[15] Piana S Ragazzi M Tallini G De Biase D Ciarrocchi A Frasoldati A. Papillary thyroid microcarcinoma with fatal outcome: evidence of tumor progression in lymph node metastases. Hum Pathology. (2013) 44:556–65. doi: 10.1016/j.humpath.2012.06.019 23079204

[16] Li F Chen G Sheng C Gusdon AM Huang Y Lv Z. BRAFV600E mutation in papillary thyroid microcarcinoma: a meta-analysis. Endocrine-Related Cancer. (2015) 22:159–68. doi: 10.1530/ERC-14-0531 PMC4629836 25593071

[17] Barbaro D Incensati RM Materazzi G Boni G Grosso M Panicucci E. The BRAF V600E mutation in papillary thyroid cancer with positive or suspected pre-surgical cytological finding is not associated with advanced stages or worse prognosis. Endocrine. (2014) 45:462–8. doi: 10.1007/s12020-013-0029-5 23925579

[18] Yang F Lian M Ma H Feng L Shen X Chen J. Identification of key genes associated with papillary thyroid microcarcinoma characteristics by integrating transcriptome sequencing and weighted gene co-expression network analysis. Gene. (2022) 811:146086. doi: 10.1016/j.gene.2021.146086 34856364

[19] Kim M Kwon CH Jang MH Kim JM Kim EH Jeon YK. Whole-exome sequencing in papillary microcarcinoma: potential early biomarkers of lateral lymph node metastasis. Endocrinol Metab. (2021) 36:1086–94. doi: 10.3803/EnM.2021.1132 PMC8566127 34731936

[20] Perera D Ghossein R Camacho N Senbabaoglu Y Seshan V Li J. Genomic and transcriptomic characterization of papillary microcarcinomas with lateral neck lymph node metastases. J Clin Endocrinol Metab. (2019) 104:4889–99. doi: 10.1210/jc.2019-00431 PMC6733494 31237614

[21] Song YS Kang BH Lee S Yoo SK Choi YS Park J. Genomic and transcriptomic characteristics according to size of papillary thyroid microcarcinoma. Cancers. (2020) 12:1345. doi: 10.3390/cancers12051345 32466217 PMC7281223

[22] Stenina-Adognravi O Plow EF. Thrombospondin-4 in tissue remodeling. Matrix Biol. (2019) 75–76:300–13. doi: 10.1016/j.matbio.2017.11.006 PMC6005712 29138119

[23] Muppala S Xiao R Krukovets I Verbovetsky D Yendamuri R Habib N. Thrombospondin-4 mediates TGF-β-induced angiogenesis. Oncogene. (2017) 36:5189–98. doi: 10.1038/onc.2017.140 PMC5589494 28481870

[24] Frolova EG Sopko N Blech L Popović ZB Li J Vasanji A. Thrombospondin-4 regulates fibrosis and remodeling of the myocardium in response to pressure overload. FASEB J. (2012) 26:2363–73. doi: 10.1096/fj.11-190728 PMC3360147 22362893

[25] Chou K Chang A Ho C Tsai T Chen H Chen P. Thrombospondin-4 promotes bladder cancer cell migration and invasion via MMP2 production. J Cell Mol Med. (2021) 25:6046–55. doi: 10.1111/jcmm.v25.13 PMC8406484 34142438

[26] Guo D Zhang D Ren M Lu G Zhang X He S. THBS4 promotes HCC progression by regulating ITGB1 via FAK/PI3K/AKT pathway. FASEB J. (2020) 34:10668–81. doi: 10.1096/fj.202000043R 32567740

[27] Shi Y Sun L Zhang R Hu Y Wu Y Dong X. Thrombospondin 4/integrin α2/HSF1 axis promotes proliferation and cancer stem-like traits of gallbladder cancer by enhancing reciprocal crosstalk between cancer-associated fibroblasts and tumor cells. J Exp Clin Cancer Res. (2021) 40:14. doi: 10.1186/s13046-020-01812-7 33407730 PMC7789630

[28] Chen X Huang Y Wang Y Wu Q Hong S Huang Z. THBS4 predicts poor outcomes and promotes proliferation and metastasis in gastric cancer. J Physiol Biochem. (2019) 75:117–23. doi: 10.1007/s13105-019-00665-9 30746617

[29] Förster S Gretschel S Jöns T Yashiro M Kemmner W. THBS4, a novel stromal molecule of diffuse-type gastric adenocarcinomas, identified by transcriptome-wide expression profiling. Modern Pathology. (2011) 24:1390–403. doi: 10.1038/modpathol.2011.99 21701537

[30] Bolger AM Lohse M Usadel B. Trimmomatic: a flexible trimmer for Illumina sequence data. Bioinformatics. (2014) 30 (15):2114–20. doi: 10.1093/bioinformatics/btu170 PMC4103590 24695404

[31] Dobin A Davis CA Schlesinger F Drenkow J Zaleski C Jha S. STAR: ultrafast universal RNA-seq aligner. Bioinformatics. (2013) 29(1):15–21. doi: 10.1093/bioinformatics/bts635 PMC3530905 23104886

[32] Li B Dewey CN. RSEM: accurate transcript quantification from RNA-Seq data with or without a reference genome. BMC Bioinf. (2011) 12:323. doi: 10.1186/1471-2105-12-323 PMC3163565 21816040

[33] Love MI Huber W Anders S. Moderated estimation of fold change and dispersion for RNA-seq data with DESeq2. Genome Biol. (2014) 15:550. doi: 10.1186/s13059-014-0550-8 25516281 PMC4302049

[34] Robinson MD McCarthy DJ Smyth GK. edgeR: a Bioconductor package for differential expression analysis of digital gene expression data. Bioinformatics. (2010) 26:139–40. doi: 10.1093/bioinformatics/btp616 PMC2796818 19910308

[35] Yu G Wang LG Han Y He QY. clusterProfiler: an R package for comparing biological themes among gene clusters. OMICS: A J Integr Biol. (2012) 16:284–7. doi: 10.1089/omi.2011.0118 PMC3339379 22455463

[36] Sturm G Finotello F List M. Immunedeconv: an R package for unified access to computational methods for estimating immune cell fractions from bulk RNA-sequencing data, in: Bioinformatics for cancer immunotherapy (2020). New York, NY: Springer US (Accessed May 2, 2024). Methods in Molecular Biology; vol. 2120. 10.1007/978-1-0716-0327-7_16 32124323

[37] Aran D Hu Z Butte AJ. xCell: digitally portraying the tissue cellular heterogeneity landscape. Genome Biol. (2017) 18:220. doi: 10.1186/s13059-017-1349-1 29141660 PMC5688663

[38] Liu J Lichtenberg T Hoadley KA Poisson LM Lazar AJ Cherniack AD. An integrated TCGA pan-cancer clinical data resource to drive high-quality survival outcome analytics. Cell. (2018) 173:400–416.e11. doi: 10.1016/j.cell.2018.02.052 29625055 PMC6066282

[39] Kim MS Choi HS Wu M Myung J Kim EJ Kim YS. Potential role of PDGFRβ-associated THBS4 in colorectal cancer development. Cancers. (2020) 12:2533. doi: 10.3390/cancers12092533 32899998 PMC7564555

[40] Kennel KB Bozlar M De Valk AF Greten FR. Cancer-associated fibroblasts in inflammation and antitumor immunity. Clin Cancer Res. (2023) 29:1009–16. doi: 10.1158/1078-0432.CCR-22-1031 PMC10011884 36399325

[41] Chen Z Zhou L Liu L Hou Y Xiong M Yang Y. Single-cell RNA sequencing highlights the role of inflammatory cancer-associated fibroblasts in bladder urothelial carcinoma. Nat Commun. (2020) 11:5077. doi: 10.1038/s41467-020-18916-5 33033240 PMC7545162

[42] Li X Sun Z Peng G Xiao Y Guo J Wu B. Single-cell RNA sequencing reveals a pro-invasive cancer-associated fibroblast subgroup associated with poor clinical outcomes in patients with gastric cancer. Theranostics. (2022) 12:620–38. doi: 10.7150/thno.60540 PMC8692898 34976204

[43] Yan T Qiu W Weng H Fan Y Zhou G Yang Z. Single-cell transcriptomic analysis of ecosystems in papillary thyroid carcinoma progression. Front Endocrinol. (2021) 12:729565. doi: 10.3389/fendo.2021.729565 PMC8591202 34790166

[44] Pu W Shi X Yu P Zhang M Liu Z Tan L. Single-cell transcriptomic analysis of the tumor ecosystems underlying initiation and progression of papillary thyroid carcinoma. Nat Commun. (2021) 12:6058. doi: 10.1038/s41467-021-26343-3 34663816 PMC8523550

[45] Isella C Terrasi A Bellomo SE Petti C Galatola G Muratore A. Stromal contribution to the colorectal cancer transcriptome. Nat Genet. (2015) 47:312–9. doi: 10.1038/ng.3224 25706627

[46] Chen Q Liu Y Jeong HW Stehling M Dinh VV Zhou B. Apelin+ Endothelial niche cells control hematopoiesis and mediate vascular regeneration after myeloablative injury. Cell Stem Cell. (2019) 25:768–783.e6. doi: 10.1016/j.stem.2019.10.006 31761723 PMC6900750

[47] Okamoto J Hirata T Chen Z Zhou HM Mikami I Li H. EMX2 is epigenetically silenced and suppresses growth in human lung cancer. Oncogene. (2010) 29:5969–75. doi: 10.1038/onc.2010.330 PMC3090446 20697358

[48] Wang L Jin J Zhou Y Tian Z He B Huang Y. EMX2 is epigenetically silenced and suppresses epithelial−mesenchymal transition in human esophageal adenocarcinoma. Oncol Rep. (2019) 29(44):5969–75. doi: 10.3892/or.2019.7284 31432154

[49] Zhan K Yang X Li S Bai Y. Correlation of endoplasmic reticulum stress patterns with the immune microenvironment in hepatocellular carcinoma: a prognostic signature analysis. Front Immunol. (2023) 14:1270774. doi: 10.3389/fimmu.2023.1270774 38143739 PMC10748430

[50] Qi L Chen J Zhou B Xu K Wang K Fang Z. HomeoboxC6 promotes metastasis by orchestrating the DKK1/Wnt/β-catenin axis in right-sided colon cancer. Cell Death Dis. (2021) 12:337. doi: 10.1038/s41419-021-03630-x 33795652 PMC8016886

[51] Ren S Wu D Shen X Wu Q Li C Xiong H. Deciphering the role of extrachromosomal circular DNA in adipose stem cells from old and young donors. Stem Cell Res Ther. (2023) 14:341. doi: 10.1186/s13287-023-03575-2 38017497 PMC10683086

[52] He S Arikin A Chen J Huang T Wu Z Wang L. Transcriptome analysis identified 2 new lncRNAs associated with the metastasis of papillary thyroid carcinoma. ORL. (2022) 84:247–54. doi: 10.1159/000518085 34818244

[53] Yang W Bai C Zhang L Li Z Tian Y Yang Z. Correlation between serum circRNA and thyroid micropapillary carcinoma with cervical lymph node metastasis. Medicine. (2020) 99:e23255. doi: 10.1097/MD.0000000000023255 33217846 PMC7676571

[54] Lee S Bae JS Jung CK Chung WY. Extensive lymphatic spread of papillary thyroid microcarcinoma is associated with an increase in expression of genes involved in epithelial-mesenchymal transition and cancer stem cell-like properties. Cancer Med. (2019) 8:6528–37. doi: 10.1002/cam4.v8.15 PMC6825983 31498560

[55] Li Q Feng T Zhu T Zhang W Qian Y Zhang H. Multi-omics profiling of papillary thyroid microcarcinoma reveals different somatic mutations and a unique transcriptomic signature. J Transl Med. (2023) 21:206. doi: 10.1186/s12967-023-04045-2 36941725 PMC10026500

[56] Frolova EG Drazba J Krukovets I Kostenko V Blech L Harry C. Control of organization and function of muscle and tendon by thrombospondin-4. Matrix Biol. (2014) 37:35–48. doi: 10.1016/j.matbio.2014.02.003 24589453 PMC4150858

[57] Gabrielsen A Lawler PR Yongzhong W Steinbrüchel D Blagoja D Paulsson-Berne G. Gene expression signals involved in ischemic injury, extracellular matrix composition and fibrosis defined by global mRNA profiling of the human left ventricular myocardium. J Mol Cell Cardiol. (2007) 42:870–83. doi: 10.1016/j.yjmcc.2006.12.016 17343875

[58] Vanhoutte D Schips TG Kwong JQ Davis J Tjondrokoesoemo A Brody MJ. Thrombospondin expression in myofibers stabilizes muscle membranes. eLife. (2016) 5:e17589. doi: 10.7554/eLife.17589 27669143 PMC5063588

[59] Liu J Cheng G Yang H Deng X Qin C Hua L. Reciprocal regulation of long noncoding RNAs THBS4-003 and THBS4 control migration and invasion in prostate cancer cell lines. Mol Med Rep. (2016) 14:1451–8. doi: 10.3892/mmr.2016.5443 PMC4940078 27357608

[60] Greco SA Chia J Inglis KJ Cozzi SJ Ramsnes I Buttenshaw RL. Thrombospondin-4 is a putative tumour-suppressor gene in colorectal cancer that exhibits age-related methylation. BMC Cancer. (2010) 10:494. doi: 10.1186/1471-2407-10-494 20846368 PMC2946314

[61] Fuyuhiro Y Yashiro M Noda S Kashiwagi S Matsuoka J Doi Y. Upregulation of cancer-associated myofibroblasts by TGF-β from scirrhous gastric carcinoma cells. Br J Cancer. (2011) 105:996–1001. doi: 10.1038/bjc.2011.330 21863023 PMC3185946

[62] Muppala S Xiao R Gajeton J Krukovets I Verbovetskiy D Stenina-Adognravi O. Thrombospondin-4 mediates hyperglycemia- and TGF -beta-induced inflammation in breast cancer. Int J Cancer. (2021) 148:2010–22. doi: 10.1002/ijc.33439 33320955

[63] Bagaev A Kotlov N Nomie K Svekolkin V Gafurov A Isaeva O. Conserved pan-cancer microenvironment subtypes predict response to immunotherapy. Cancer Cell. (2021) 39:845–865.e7. doi: 10.1016/j.ccell.2021.04.014 34019806

[64] Yang Z Wei X Pan Y Xu J Si Y Min Z. A new risk factor indicator for papillary thyroid cancer based on immune infiltration. Cell Death Dis. (2021) 12:51. doi: 10.1038/s41419-020-03294-z 33414407 PMC7791058

[65] Minna E Brich S Todoerti K Pilotti S Collini P Bonaldi E. Cancer associated fibroblasts and senescent thyroid cells in the invasive front of thyroid carcinoma. Cancers. (2020) 12:112. doi: 10.3390/cancers12010112 31906302 PMC7016563

[66] Tsoumakidou M. The advent of immune stimulating CAFs in cancer. Nat Rev Cancer. (2023) 23:258–69. doi: 10.1038/s41568-023-00549-7 36807417

[67] Chen Y McAndrews KM Kalluri R. Clinical and therapeutic relevance of cancer-associated fibroblasts. Nat Rev Clin Oncol. (2021) 18:792–804. doi: 10.1038/s41571-021-00546-5 34489603 PMC8791784

[68] Chen X Song E. Turning foes to friends: targeting cancer-associated fibroblasts. Nat Rev Drug Discovery. (2019) 18:99–115. doi: 10.1038/s41573-018-0004-1 30470818

[69] Hanahan D. Hallmarks of cancer: new dimensions. Cancer Discovery. (2022) 12:31–46. doi: 10.1158/2159-8290.CD-21-1059 35022204

[70] Sahai E Astsaturov I Cukierman E DeNardo DG Egeblad M Evans RM. A framework for advancing our understanding of cancer-associated fibroblasts. Nat Rev Cancer. (2020) 20:174–86. doi: 10.1038/s41568-019-0238-1 PMC7046529 31980749

[71] Woo CG Sung CO Choi YM Kim WG Kim TY Shong YK. Clinicopathological significance of minimal extrathyroid extension in solitary papillary thyroid carcinomas. Ann Surg Oncol. (2015) 22:728–33. doi: 10.1245/s10434-015-4659-0 PMC4686556 26077913

