# Supplementary figures and images for: Transcriptome sequencing revealed that lymph node metastasis of papillary thyroid microcarcinoma is associated with high THBS4 expression and PDGFRA+ cancer-associated fibroblasts
Source: Front Oncol. 2025 Apr 15;15:1536063. doi: 10.3389/fonc.2025.1536063 (PMC12037473; doi:10.3389/fonc.2025.1536063)

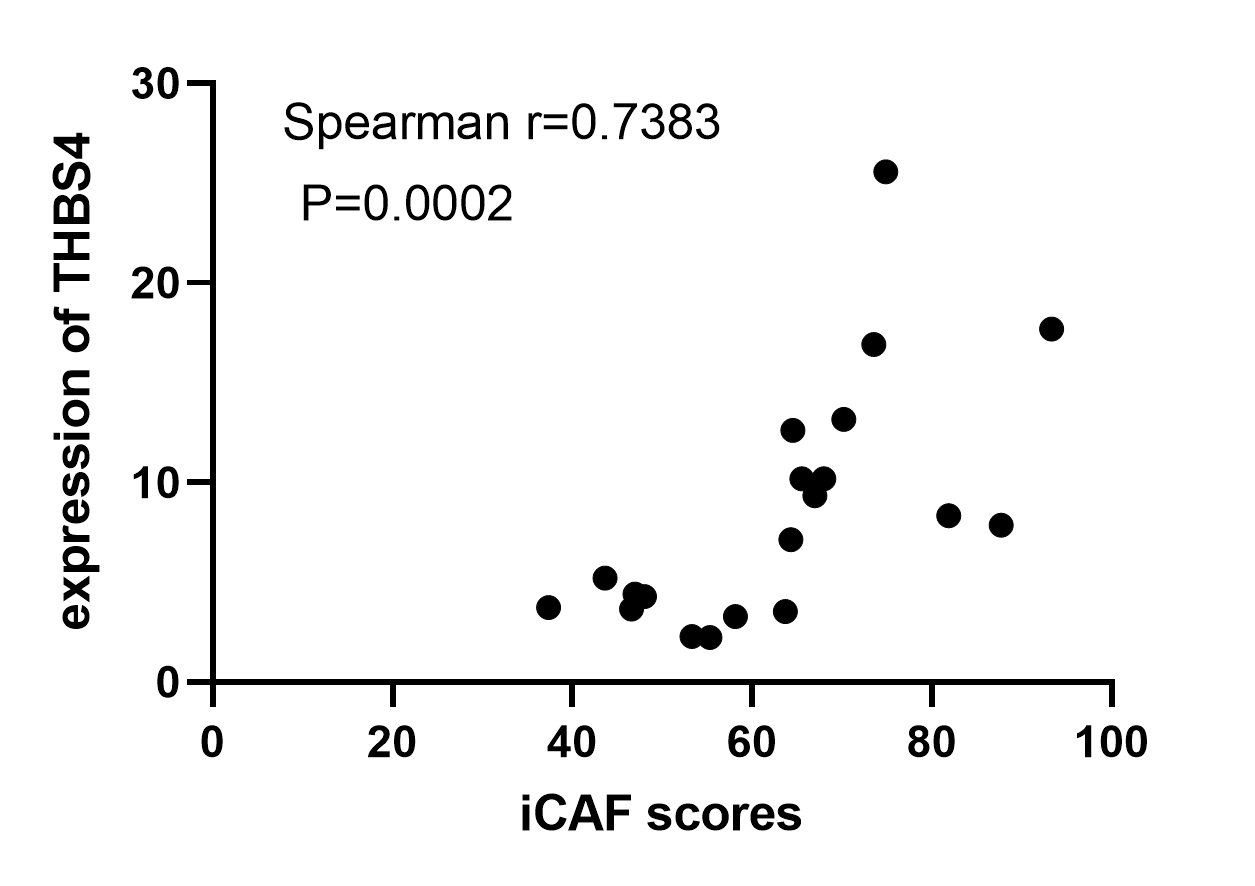

Supplement: Supplementary Figure 1 — Correlation analysis of expression of THBS4 and iCAF scores, R=0.738, p<0.001, Spearman rank correlation [file DataSheet1.zip › Figure S1.tif]
