# Supplementary material for: Transcriptome sequencing revealed that lymph node metastasis of papillary thyroid microcarcinoma is associated with high THBS4 expression and PDGFRA+ cancer-associated fibroblasts
Source: Front Oncol. 2025 Apr 15;15:1536063. doi: 10.3389/fonc.2025.1536063 (PMC12037473; doi:10.3389/fonc.2025.1536063)
Supplement: Supplementary Figure 1 — Correlation analysis of expression of THBS4 and iCAF scores, R=0.738, p<0.001, Spearman rank correlation [file DataSheet1.zip › Table S1 and S2.doc]

**Table S1 Top marker genes for iCAF and myoCAF in PTC**

| **iCAF markers** | **myoCAFmarkers** |
| --- | --- |
| CFD | RGS5 |
| MFAP5 | NDUFA4L2 |
| SFRP4 | ACTA2 |
| DCN | FABP4 |
| PLA2G2A | HIGD1B |
| IGFBP6 | TPM2 |
| MFAP4 | COX4I2 |
| FBLN1 | MEF2C |
| SFRP2 | CD36 |
| CCDC80 | TINAGL1 |
| APOD | PPP1R14A |
| PI16 | APOE |
| CXCL14 | ARHGDIB |
| S100A10 | GJA4 |
| IGFBP5 | A2M |
| TNXB | MYLK |
| FBN1 | MYL9 |
| PRG4 | PGF |
| FBLN2 | COL18A1 |
| ACKR3 | STEAP4 |
| SLPI | FABP5 |
| EFEMP1 | TAGLN |
| PCOLCE2 | ADIRF |
| ITM2A | COL4A2 |
| C1S | CHN1 |

**Table.S2 Differentially expressed genes encoding Long non-coding RNAs**

| **Gene** | **logFC** | **P-value** | **Adj-P** | **Regulation** | **Gene_symbol** |
| --- | --- | --- | --- | --- | --- |
| ENST00000429922.1 | 5.204337 | 6.68E-10 | 3.10E-06 | Up | LENG8-AS1 |
| ENST00000548801.1 | 1.577164 | 0.000211 | 0.489046 | Up | MIR10A |
| ENST00000551288.1 | 2.657302 | 0.000422 | 0.490089 | Up | EMX2OS |
| MSTRG.14409.1 | -2.43305 | 0.00039 | 0.490089 | Down |  |
| ENST00000369381.2 | 3.513819 | 0.000635 | 0.500373 | Up | RP11-417J8.6 |
| ENST00000448179.1 | 2.244259 | 0.001167 | 0.774422 | Up | RP11-54O7.1 |
| ENST00000519691.1 | 3.399713 | 0.002109 | 0.990634 | Up | RP11-150O12.3 |
| ENST00000360902.1 | -4.01794 | 0.006235 | 0.99857 | Down | AC004893.11 |
| ENST00000380722.1 | 1.195425 | 0.01222 | 0.99857 | Up | RP11-497E19.1 |
| ENST00000414120.1 | 1.484945 | 0.039368 | 0.99857 | Up | LINC00887 |
| ENST00000417071.1 | 2.953771 | 0.044261 | 0.99857 | Up | TTTY15 |
| ENST00000433108.1 | 1.574902 | 0.033609 | 0.99857 | Up | MIR205HG |
| ENST00000433152.4 | 1.163952 | 0.007945 | 0.99857 | Up | RP11-222A11.1 |
| ENST00000434301.1 | -1.33102 | 0.031321 | 0.99857 | Down | AC092667.2 |
| ENST00000439707.2 | 1.826843 | 0.032962 | 0.99857 | Up | LINC00284 |
| ENST00000441212.1 | -1.31962 | 0.01452 | 0.99857 | Down | AC078883.4 |
| ENST00000442329.2 | 1.417939 | 0.005746 | 0.99857 | Up | TTN-AS1 |
| ENST00000448587.1 | 1.911823 | 0.013435 | 0.99857 | Up | TINCR |
| ENST00000458145.1 | -1.18053 | 0.013769 | 0.99857 | Down | RP11-90C4.2 |
| ENST00000468646.2 | -2.2999 | 0.026957 | 0.99857 | Down | LINC00877 |
| ENST00000501938.2 | 1.856536 | 0.041231 | 0.99857 | Up | RGMB-AS1 |
| ENST00000505532.1 | 1.811971 | 0.018669 | 0.99857 | Up | RP11-597D13.9 |
| ENST00000507446.1 | 3.593186 | 0.01181 | 0.99857 | Up | RP11-440L14.1 |
| ENST00000508031.1 | -1.30851 | 0.039397 | 0.99857 | Down | RP11-44F21.3 |
| ENST00000508671.1 | -2.11846 | 0.032758 | 0.99857 | Down | RP11-693J15.4 |
| ENST00000514843.1 | -1.04148 | 0.005495 | 0.99857 | Down | RP11-76G10.1 |
| ENST00000517550.1 | 1.265166 | 0.042841 | 0.99857 | Up | HOXA-AS2 |
| ENST00000528549.1 | 2.195276 | 0.005581 | 0.99857 | Up | TBX5-AS1 |
| ENST00000536317.1 | -1.4905 | 0.020476 | 0.99857 | Down | RP11-1060J15.4 |
| ENST00000540906.1 | 1.389264 | 0.00688 | 0.99857 | Up | RP11-794G24.1 |
| ENST00000544925.1 | -1.61114 | 0.017886 | 0.99857 | Down | RP11-64D24.2 |
| ENST00000551729.1 | -1.14451 | 0.021181 | 0.99857 | Down | RP11-1022B3.1 |
| ENST00000553633.1 | 1.154824 | 0.012551 | 0.99857 | Up | RP11-840I19.3 |
| ENST00000556405.1 | -1.64949 | 0.004718 | 0.99857 | Down | RP11-398E10.1 |
| ENST00000559203.1 | -1.11764 | 0.005634 | 0.99857 | Down | RP11-219B17.1 |
| ENST00000561811.1 | -1.58691 | 0.016864 | 0.99857 | Down | RP11-61A14.3 |
| ENST00000562866.1 | 1.285514 | 0.007842 | 0.99857 | Up | VPS9D1-AS1 |
| ENST00000563424.2 | 1.252302 | 0.004631 | 0.99857 | Up | RP11-401P9.4 |
| ENST00000585285.1 | 1.063065 | 0.023165 | 0.99857 | Up | MIR3615 |
| ENST00000585798.1 | 1.216942 | 0.018742 | 0.99857 | Up | RP11-13K12.5 |
| ENST00000588717.1 | 1.041681 | 0.029302 | 0.99857 | Up | CTD-2162K18.3 |
| ENST00000590433.1 | -1.16277 | 0.026979 | 0.99857 | Down | CTD-2554C21.2 |
| ENST00000593438.1 | 1.568396 | 0.003393 | 0.99857 | Up | HOXA-AS2 |
| ENST00000595010.1 | -1.01201 | 0.009124 | 0.99857 | Down | HCCAT3 |
| ENST00000598996.1 | 1.257031 | 0.027857 | 0.99857 | Up | FENDRR |
| ENST00000602471.1 | -1.5219 | 0.048477 | 0.99857 | Down | CTD-2306M10.1 |
| ENST00000602595.1 | -1.0526 | 0.010057 | 0.99857 | Down | RP11-932O9.8 |
| ENST00000602957.1 | 1.579084 | 0.008529 | 0.99857 | Up | RP6-91H8.3 |
| ENST00000606336.1 | 1.052273 | 0.040366 | 0.99857 | Up | CASC15 |
| ENST00000607746.1 | -2.16592 | 0.002798 | 0.99857 | Down | RP11-861A13.4 |
| ENST00000608774.1 | -1.39167 | 0.005641 | 0.99857 | Down | RP11-188P17.2 |
| ENST00000610122.1 | -1.94124 | 0.023072 | 0.99857 | Down | RP11-1275H24.1 |
| MSTRG.66649.1 | -1.05232 | 0.015691 | 0.99857 | Down |  |
| MSTRG.719.1 | 1.343654 | 0.022483 | 0.99857 | Up |  |
| MSTRG.9534.2 | -3.92094 | 0.027143 | 0.99857 | Down |  |
